# Deceived by stripes: conspicuous patterning on vital anterior body parts can redirect predatory strikes to expendable posterior organs

**DOI:** 10.1098/rsos.160057

**Published:** 2016-06-08

**Authors:** Gopal Murali, Ullasa Kodandaramaiah

**Affiliations:** School of Biology, Indian Institute of Science Education and Research Thiruvananthapuram, CET campus, Trivandrum 695016, India

**Keywords:** stripes, deflection, redirection hypothesis, motion perception, motion dazzle, lacertilians

## Abstract

Conspicuous coloration, which presumably makes prey more visible to predators, has intrigued researchers for long. Contrastingly coloured, conspicuous striped patterns are common among lizards and other animals, but their function is not well known. We propose and test a novel hypothesis, the ‘redirection hypothesis’, wherein longitudinal striped patterns, such as those found on the anterior body parts of most lacertilians, redirect attacks away from themselves during motion towards less vulnerable posterior parts, for example, the autotomous tail. In experiments employing human ‘predators’ attacking virtual prey on a touchscreen, we show that longitudinal striped patterns on the anterior half of prey decreased attacks to the anterior and increased attacks to the posterior. The position of stripes mattered—they worked best when they were at the anterior. By employing an adaptive psychophysical procedure, we show that prey with striped patterning are perceived to move slower, offering a mechanistic explanation for the redirective effect. In summary, our results suggest that the presence of stripes on the body (i.e. head and trunk) of lizards in combination with caudal autotomy can work as an effective anti-predator strategy during motion.

## 1. Introduction

A striking feature of several thousands of lizards (Suborder: Lacertilia), snakes (Suborder: Serpents) and other animals is the presence of strongly contrasting stripes on the body, which presumably make them conspicuous against their background and hence enhance detection by predators. Researchers have, for long, attempted to explain the evolutionary significance of such conspicuous colour patterns, especially in the context of prey–predator signalling [1–5]. Recent studies have shown that conspicuous high-contrast patterns such as stripes may play a role against predation during motion [6–8], supporting the ‘motion dazzle hypothesis’ [4]. This hypothesis posits that during motion, the presence of high-contrast patterns interferes with precise estimation of speed or trajectory of the prey by the predator, thereby reducing the likelihood of successful capture. Despite a few contradictory results [9,10], the majority of experimental and comparative studies [3,11–15] on this topic has suggested that motion dazzle patterns might create erroneous motion signals, causing an apparent change in the perceived speed by the predator (see [8] for a review on mechanisms).

**Figure 1.**
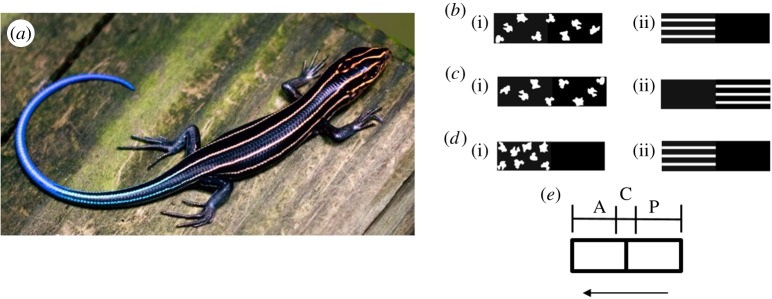
(*a*) Example of a scincid lizard (*Plestiodon japonicus*) with striped body coloration (photo credit: Kris Kelley via Wikimedia Commons), (*b*–*d*) prey used in the experiments (*b*) Experiment 1: (i) control (C), (ii) stripe-anterior (S-A); (*c*) Experiment 2: (i) control (C), (ii) stripe-posterior (S-P); (*d*) Experiment 3: (i) control-anterior (C-A), (ii) stripe-anterior (S-A), (*e*) hypothetical prey labelled with different parts; anterior (A), centre (C) and posterior (P), the arrow represents direction of prey movement; for simplicity only prey from *Set* 1 are shown.

A large proportion of lacertilian (lizard) species have contrasting stripes that run parallel to the body and therefore the direction of motion (hereafter longitudinal stripes; figure 1*a*). Studies have focused on correlations between longitudinal stripes and factors such as escape strategy [16,17], morphometrics [18,19], fitness proxies [18] and activity patterns [19]. A special feature of striped markings in most striped lacertilians is that they are localized on the trunk, while the tail is plainly coloured (figure 1*a*; electronic supplementary material, figure S10). Currently, there is no evidence that anterior longitudinal stripes in lizards play a role against predation. On the other hand, a few studies have suggested that longitudinal striped coloration may have no benefit whatsoever [9,20].

Another widely employed defence strategy is deflection, wherein markings on the prey increase the propensity of a predator's initial strike to less-vital body parts such as the wing margin of a butterfly or the fin of a fish [2,21,22]. The classical deflective markings against visually oriented predators include eyespots [23,24] and other conspicuously coloured patterns [25,26], which have received much research attention recently. For instance, it has been demonstrated using stationary models mimicking lizards that attacks are deflected to the ‘autotomous’ tail when the tail is conspicuously coloured and in stark contrast with the body colour of the lizards [27,28] or behaviourally by waving the tail to attract the predator's initial attack [29]. Therefore, there is strong experimental evidence that certain body patterns can *attract* attention *towards* themselves, thereby deflecting attacks away from vital body parts.

Alternatively, markings can theoretically also function by *redirecting* attacks *away* from themselves by providing misleading motion cues [24,30]. However, until now, no experimental study has tested the influence of colour markings found on vulnerable anterior body parts such as the head or trunk of an animal in deflecting attacks away from themselves. Moreover, studies involving motion dazzle patterns have neglected the positioning of patterns on the body of real animals. It is possible that the effect produced by motion dazzle patterns (i.e. perceived speed difference) might be influenced by the localization of these patterns on the body, especially in relation to escape direction.

We here test a novel hypothesis: longitudinal striped patterns, such as those found on the anterior body parts of most lacertilians, redirect attacks away from themselves and towards the less vulnerable, autotomous tail through the ‘motion dazzle’ effect. We tested the effect of stripes on the location of strikes in a series of experiments by using human volunteers as test subjects, who were instructed to attack moving ‘prey’ objects on a touch screen computer. We show that prey with striped patterns on the anterior half received attacks more often to posterior half compared with suitable controls. By employing an adaptive staircase paradigm [9,31], we also demonstrate that stripes on the anterior part of the body (we hereafter use the term body to mean the anterior part of the lizard, i.e. head and trunk) make the prey appear to move slower, thus providing a mechanistic explanation for the observed redirective effect.

## 2. Material and methods

### 2.1. Experimental design

#### 2.1.1. Redirection experiments (Experiments 1–3)

The experiments involved a game written in the software SCRATCH 2.0 (2014), where a single-patterned ‘prey’ entered the display from one of the four edges and exited from another, with the entrance and exit edges chosen randomly. The black and white rectangular prey, effectively 3 × 0.9 cm (visual angle: 2.644 × 0.793°) each on the display screen, were generated using the software Inkscape 0.48 (2010). The proportion of black (86.4%) and white (13.6%) remained constant across all prey. Depending on the treatment, prey had (i) alternating white and black stripes, either on the anterior or on the posterior halves or (ii) irregular blotched white patterns on a black background, on the anterior and/or the posterior halves (figure 1*b–d*). In all experiments, the same irregular blotched white patterns were used. The prey moved in a random manner with an approximate speed of 17.4390 cm s^−1^ (15.281 visual angles s^−1^) on the display area of dimensions 20 × 27 cm (visual angle: 17.492 × 23.466°; refer to electronic supplementary material, Text S1 for detailed descriptions of methods related to prey behaviour). To discriminate which half of the prey was attacked, the *black* on the anterior and posterior halves of each prey was assigned different colours: In *Set* 1, consisting of half of the samples within a treatment, the anterior black was coded as ‘pure-black’ (RGB: 0,0,0) and the posterior black as ‘greyish-black’ (RGB: 20,20,20). In *Set* 2 ‘pure-black’ and ‘greyish-black’ were switched (electronic supplementary material, table S1). Each experiment consisted of two prey types (treatment and control), with each prey type having two *Sets*.

The experiments were conducted on the display of an HP Envy 17.3-inch Touch-Smart laptop. A total of 155 volunteers, all of whom were naive to the experimental hypothesis, were used. No participant was used in more than one trial. Prior to the start of each trial, subjects were given written instructions (see the electronic supplementary material S2). In brief, volunteers were instructed to touch the anterior half (in relation to the direction of movement) of the prey. Each trial entailed the same individual attacking two prey (treatment and control) sequentially for a minute each. The order of presentation of prey was alternated between trials in order to eliminate bias due to presentation order. A touch on a prey was scored as an attack and recording of the number of attacks at the *anterior*, *posterior* and *centre* (touching both colours) was automated (figure 1*e*). A circle of diameter 0.3 cm (0.264°) was used as a ‘touch sensor’ to record the number of attacks.

Experiment 1 tested the redirective effect of striped patterns by comparing attacks on prey with stripes on the anterior half (S-A; figure 1*b*) to that on prey with irregular blotched patterns on both halves (C; figure 1*b*). If striped patterns redirect attacks to the posterior, a greater proportion of attacks must be directed to the posterior half in the case of S-A. Experiment 2 compared prey with longitudinal stripes on the posterior half (S-P; figure 1*c*), i.e. the reverse of S-A as in Experiment 1 and control (C; same as in Experiment 1) to test whether the position of stripes on the prey influences the redirective effect. Experiment 3 tested whether highly contrasting patterns *per se* on the anterior half of the prey had a redirective effect. This was done by comparing prey with plain posterior halves and with the anterior having either stripes (S-A; figure 1*d*) or irregular blotched patterns (C-A; figure 1*d*). Full details of the experimental set-up, game, stimulus-design and procedures are given in the electronic supplementary material, Text S1).

#### 2.1.2. Speed match experiment (Experiment 4)

This experiment involved another SCRATCH interactive game designed to test whether the perceived speeds differed between stimuli with striped and blotched patterns. The stimuli used in the experiment were the same as in Experiment 1 (S-A and C figure 1*b*). Participants (*n* = 55) were asked to compare the speed of the stimuli—‘standard’ and ‘target’—presented one after another. A randomly chosen stimulus (i.e. either S-A or C) moved across the display from the bottom edge with an entry angle in the range of −30^o^ to +30^o^ (normalized to the vertical axis) until it touched one of the three other edges, when it disappeared. The same stimulus appeared and disappeared once more. This was followed by an interval of 1.5 s, after which the second stimulus similarly moved twice across the display. Both stimuli were presented twice as our pilot experiments indicated that speed judgement was difficult when the prey was viewed once (refer to electronic supplementary material, Text S1 for more details). After the disappearance of both stimuli, a question was projected on the screen (electronic supplementary material, figure S9) and participants were asked to choose one among the three options—(i) *target* was faster, (ii) *standard* was faster or (iii) both were equally fast. Their choice was registered by a touch on one of the three options (electronic supplementary material, figure S9).

An adaptive staircase procedure from von Helverson *et al.* [9] was adapted for speed comparisons. In the first set-up, the striped stimulus (S-A) was chosen as the *standard* and the control (C) as the *target s*timulus, whereas this was switched in the second set-up. The speed of the *standard* was kept constant (17.4390 cm s^−1^) throughout the experiment, but the speed of the *target* was modified in trial *n* *+* 1 based on the response of the participant in trial *n*. For example, if participant responded that the *target* moved faster, then the speed of *target* was decreased by 0.35 cm s^−1^ in the next trial, and the decrease continued through the next trials until the participant indicated that it was now slower than the *standard* stimulus. If the response was *equal*, then the speed was incremented or decremented by 0.35 cm s^−1^ based on the direction of previous response. For example, the speed was increased if previous response resulted in increase of speed. The speed-matched values for first six reversal events (i.e. number of times the target is perceived to move from slower to faster or faster to slower) were considered for the analysis. The matched speeds of the *target* indicate the actual speed of the *target* when it is perceived to move at the same speed as the *standard* (for illustrative graph, see the electronic supplementary material, figure S7). The *target* was presented with three different initial speeds 15.8456, 17.4390 and 19.3888 cm s^−1^. The order of presentation of the stimulus and the initial speed condition was chosen randomly for each presentation.

#### 2.1.3. Statistical analyses

All analyses were performed in RStudio v 3.0.3 ‘Warm Puppy’ [32]. Under natural conditions, it is expected that attacks to the head or trunk of an animal are lethal. We therefore pooled counts for the number of hits at the *anterior* and *centre* and considered the sum as the number of lethal attacks (*n*_lethal_) and number of attacks to *posterior* as non-lethal (*n*_non-lethal_). We analysed the data using generalized linear mixed-effects model (GLMM) using the *glmer* function in the *lme4* package [33], with counts of *n*_lethal_ and *n*_non-lethal_ as binomial-dependent variables (using *cbind* function) with *logit* link function. In all the models, prey type and presentation order (to account for learning) were included as fixed effects, while subject ID and *Set* was included as random intercept terms. Outliers (*n* = 6) with fewer attacks (less than or equal to 3) to either *anterior* or *posterior* were not included for the final analysis as their exclusion significantly improved the fit of the model (all ΔAIC > 20).

The starting model contained all the above fixed effects and possible interaction terms, which was then simplified by backward stepwise elimination of non-significant terms using the likelihood-ratio test (LRT) to obtain the model with the lowest AIC. Furthermore, for each experiment we also tested whether there was a difference in a total number of hits (*anterior* *+* *centre* *+* *posterior*) between treatments, using GLMM with Poisson error distribution, with the same fixed and random effects as that of binomial GLMM. The control (C or C-A) was considered as the reference level in all analyses. All possible pairwise comparisons for the interaction between prey type and presentation order for the Poisson GLMM were calculated using the *lsmeans* function in the *lsmeans* package [34] using the *Tukey* method. The stepwise model selection results for both binomial and Poisson GLMM methods are available in the electronic supplementary material, tables S7 and S8.

The speed match data were analysed using a linear-mixed model (LMM) with subject ID as random effect (random intercept) and initial speed, target type as fixed effects against speed matched values (average speed at which participant perceived both stimuli to move at equal speed). Refer to the electronic supplementary material, table S10 for details on model selection.

## 3. Results

### 3.1. Redirection experiments

#### 3.1.1. Experiment 1

**Figure 2.**
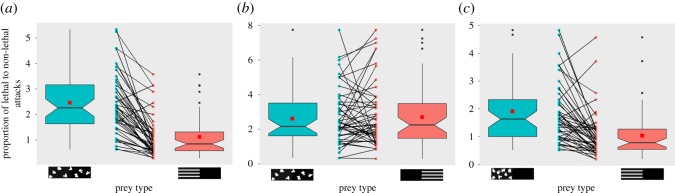
Boxplots showing proportions of lethal (anterior + centre) to non-lethal attacks (posterior) with corresponding prey type (*a*) Experiment 1: proportion greater for control (C) (binomial GLMM (*n* = 48): *z* = −12.497, *p* < 0.001) (*b*) Experiment 2: both prey receiving equal proportion of attacks (binomial GLMM (*n* = 52): *χ*^2^ = 0.0291, *p* = 0.6475) (*c*) Experiment 3: proportion greater for control (C-A) (binomial GLMM (*n* = 49): *z* = −9.884, *p* < 0.001); means are represented by red squares and paired dots across prey type represent subject-wise responses.

The treatment with stripes at the anterior (S-A) received proportionally fewer lethal to non-lethal attacks compared with the control (C) (binomial GLMM (*n* = 48): *z* = −12.497, *p* < 0.001; figure 2*a* and table 1*a*). When prey with stripes were presented first, both striped (S-A) and control prey (C) received a lower proportion of lethal to non-lethal attacks compared with when the control (C) was presented first (binomial GLMM (*n* = 48): *z* = −2.064, *p* = 0.039; table 1*a*).

**Table 1.** Results of the binomial GLMM analysis for (*a*) Experiment 1 and (*b*) Experiment 3 with the ratio of lethal attacks (*anterior* *+* *centre*) to non-lethal attacks (*posterior*) as the dependent variable and the following as fixed effects: presentation order, prey type and interaction between prey type and presentation order. *Set* number and subject ID were included as random intercept terms. Only the best fit models are presented. Bold text and * indicates significance at *p* < 0.05 and *** indicates significance at *p* < 0.0001. Details of the stepwise elimination terms are presented in electronic supplementary material, table S7.

factor	estimate	s.e.	*z*-value	*p*-value
(*a*) Experiment 1				
(intercept)	0.9031	0.11333	7.970	1.59 × 10^−15^***
prey type	−0.8733	0.0698	−12.497	<2 × 10^−16^***
**presentation order**	**−0.3009**	**0.1458**	**−2.064**	0.039*
(*b*) Experiment 3				
(intercept)	0.4491	0.0801	5.603	2.11 × 10^−8^***
prey type	−0.6562	0.0663	−9.884	<2 × 10^−16^***

**Figure 3.**
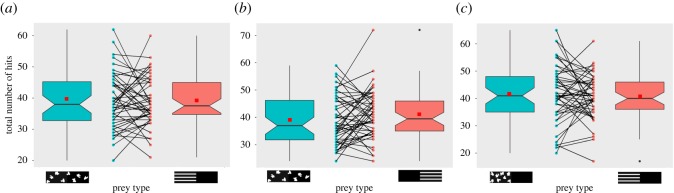
Boxplots showing total number of hits with corresponding prey types in (*a*) Experiment 1: greater number of total hits on the striped prey (S) in relation to that on the control (C) (Poisson GLMM (*n* = 48): *z* = 1.97, *p* = 0.0484), (*b*) Experiment 2: no difference between control and striped prey (Poisson GLMM (*n* = 52): *χ*^2^ = 2.6576, *p* = 0.1031), (*c*) Experiment 3: no difference between the two prey (Poisson GLMM (*n* = 49): *z* = 1.52, *p* = 0.1292); means are represented by red squares and paired dots across prey type represent subject-wise responses.

The prey with stripes (S-A) received a marginally greater total number of hits when compared to control (C) (Poisson GLMM (*n* = 48): *z* = 1.97, *p* = 0.0484; figure 3*a*; electronic supplementary material, table S9 (a)). Presentation order (Poisson GLMM (*n* = 48): *z* = 3.35, *p* = 0.0004; electronic supplementary material, table S9 (a)) and the interaction between presentation order and prey type were also significant (Poisson GLMM (*n* = 48): *z* = −3.04, *p* = 0.0023). The control prey (C) when presented first received significantly fewer total hits compared to when presented after the striped prey (S-A) (*z* ratio = −3.5283, *p* = 0.0024; electronic supplementary material, table S4 and figure S5).

#### 3.1.2. Experiment 2

The proportion of lethal to non-lethal attacks did not differ between the prey containing stripes at the posterior (S-P) and the control (C) (LRT (*n* = 52): *χ*^2^ = 0.0291, *p* = 0.6475; figure 2*b*; electronic supplementary material, table S7 (b)). There was no significant effect of presentation order (LRT (*n* = 52): *χ*^2^ = 1.5133, *p* = 0.2189) on the proportion of lethal to non-lethal attacks.

Prey with stripes at the posterior (S-P) received an equal number of total hits when compared with the control (C) (LRT (*n* = 52): *χ*^2^ = 2.6576, *p* = 0.1031; figure 3*b*; electronic supplementary material, table S8 (b)). There was no significant effect of presentation order (LRT (*n* = 52): *χ*^2^ = 0.2114, *p* = 0.6433) or interaction between presentation order and prey type (LRT (*n* = 52): *χ*^2^ = −2.9142, *p* = 0.0878).

#### 3.1.3. Experiment 3

The prey containing striped patterning at the anterior (S-A) received a lower proportion of lethal to non-lethal attacks compared with prey with irregular blotches (C-A) (binomial GLMM (*n* = 49): *z* = −9.884, *p* < 0.001; figure 2*c*, table 1*b*). Presentation order did not influence the proportion of lethal to non-lethal attacks (LRT (*n* = 49): *χ*^2^ = 0.2213, *p* = 0.6381).

The total number of hits did not differ between the treatment with stripes (S-A) and control (C-A) (Poisson GLMM (*n* = 49): *z* = 1.52, *p* = 0.1292; figure 3*c*; electronic supplementary material, table S8 (c)). Presentation order did not have any effect on the total number of hits (Poisson GLMM (*n* = 49): *z* = 1.37, *p* = 0.1716), while the interaction between presentation order and prey type was significant (Poisson GLMM (*n* = 49): *z* = −3.11, *p* = 0.0030). When striped prey (S-A) was presented first, the total number of hits for control prey (C-A) was significantly higher when compared with striped prey (S-A) (*z*-ratio = 2.6808, *p* = 0.0370; electronic supplementary material, table S4 and figure S6).

### 3.2. Speed match experiment

#### 3.2.1. Experiment 4

**Figure 4.**
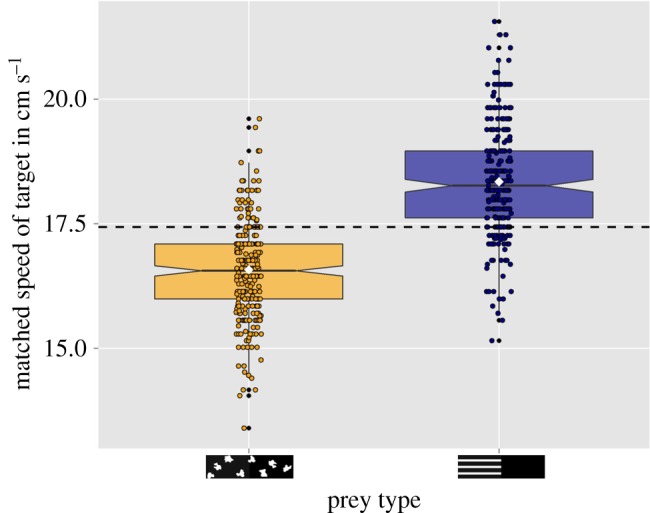
Boxplots showing the matched speed of the target stimulus: when the striped stimulus was set as the standard, the speed of control (target) was lowered (mean ± s.e.: 16.5790 ± 0.9741 cm s^−1^) to match the speed of striped stimulus (fixed speed: 17.43907 cm s^−1^). When the striped stimulus was set as the target, the matched speed of target was increased (mean ± s.e.: 18.35049 ± 1.1011 cm s^−1^) to match the speed of the non-striped standard (fixed speed: 17.4390 cm s^−1^). Means are represented by white rhombuses. The dotted line represents the speed of standard used in the experiment (i.e. control and striped stimulus for striped and control prey, respectively).

When the striped stimulus (S-A) was set as *standard*, the matched speed of the *target* (control stimulus-C) was significantly lower than that of the *standard* (LMM (*n* = 28): *t* = −5.65, *p* < 0.001; figure 4 and table 2*b*). There was no significant effect of initial speed on the matched speed (LRT (*n* = 28): *χ*^2^ = 0.5114, *p* = 0.7744) and neither was there an interaction effect between initial speed and stimulus type (LMM (*n* = 28): *χ*^2^ = 0.3132, *p* = 0.8550). When the control stimulus (C) was set as *standard*, the matched speed of the *target* (striped stimulus-S-A) was significantly greater than that of the control (LMM (*n* = 27): *t* = 4.92, *p* < 0.001; figure 4 and table 2*a*). However, there was no significant effect of the initial speed on the matched speed (LMM (*n* = 27): *χ*^2^ = 2.0334, *p* = 0.3618) or interaction between the two (LRT (*n* = 27): *χ*^2^ = 0.8846, *p* = 0.6426).

**Table 2.** Results of best fit LMM model on speed match data from Experiment 4 when (*a*) control (C) was set as standard and (*b*) striped prey (S-A) was set as standard. Speed match values were set as the dependent variable. Initial speed, prey type and interaction between prey type and initial speed were included as fixed effects. Subject ID was included as the random intercept term. Only the best-fit models are presented. Details on stepwise elimination of terms are presented in electronic supplementary material, table S10.

factor	estimate	s.e.	*t*-value	*p*-value
(*a*) control (C) as standard				
(intercept)	18.3469	0.1158	158.41	<0.0001
prey type	−0.9079	0.1846	−4.92	<0.0001
(*b*) striped prey (S-A) as standard				
(intercept)	17.4391	0.1766	98.77	<0.0001
prey type	−0.8588	0.1519	−5.65	<0.0001

## 4. Discussion

It is clear that the proportion of lethal attacks (i.e. on the *anterior* and *centre* combined) in relation to non-lethal attacks (i.e. on the *posterior*) was significantly reduced when the anterior part was striped compared with when irregular blotched patterns were present either (i) along the entire length (Experiment 1) or (ii) only on the anterior (Experiment 3; figure 2). Conversely, results from Experiment 2 suggest that stripes on the posterior half (S-P) were not beneficial in terms of reducing the lethal attacks compared to the control (C). The total number of hits (i.e. *anterior* *+* *centre* *+* *posterior*) did not differ between prey in Experiments 2 and 3, whereas striped prey (S-A) received a marginally higher number of attacks in Experiment 1 (*p* = 0.048; figure 3). However, analyses based on the number of lethal and non-lethal attacks (electronic supplementary material, tables S1, S2 and figures S1, S2) mirrored the results based on the proportion of lethal to non-lethal attacks for all three experiments, wherein stripes on the anterior (S-A) decreased the number of lethal attacks when compared with stripes on the posterior (S-P) or blotched patterns (C or C-A).

On comparing *n*_lethal_/*n*_non-lethal_ across the control prey from the three experiments ((i) with blotched patterns in both anterior and posterior (C), in Experiments 1 and 2 and (ii) with blotched patterns only in anterior (C-A); Experiment 3; electronic supplementary material, table S5 and figure S3), we found that blotched patterns in the anterior (C-A) were also effective in reducing the attacks to the anterior half, validating the results of a previous study [10]. However, the stronger redirective effect of striped prey (S-A; stripes only in the anterior) in Experiment 3 over control prey (C-A; blotched patterns only in the anterior) indicates that stripes are better at redirecting attacks compared with blotched patterns. In summary, our results clearly demonstrate that longitudinal stripes on the anterior part of the body redirect attacks towards the posterior, thereby enhancing the probability of survival. Moreover, lizard tails can be easily regenerated [35] and individuals with regenerated tails are commonly encountered in the wild (observed by the authors on many occasions). The ubiquitous presence of striped patterning on the head and trunk of lizards, which is expected to increase detection by predators, has hitherto not been convincingly explained. Our study provides strong evidence for the role of the ubiquitous striped patterning in lizards in an anti-predator context.

### 4.1 Influence of striped coloration on speed perception

One explanation for the redirective effect is that a prey with stripes on the anterior is perceived to move slower than its actual speed. Thus, a predator aiming to attack the anterior might be deceived into attacking the posterior body parts. It has been unclear whether striped objects are perceived to move faster or slower, as evidence for both exists in psychophysics literature [9,36–38]. However, the results from the speed match experiment strongly support the idea that prey with stripes (S-A) are perceived to move slower than the control (C). Striped stimuli (S-A) had a lower matched speed compared with the control (C), both when the control was used as the ‘standard’ and striped stimulus as the ‘target’, or vice versa (i.e. with the control as ‘target’; figure 4). Our study hence provides a mechanistic explanation for the observed redirective effect.

The above findings complement previous work, suggesting that striped patterns might function similar to other high-contrast motion dazzle patterns, i.e. by causing a reduction in perceived speed [14]. However, Helversen *et al.* [9] using a human predation experiment similar to ours concluded that striped patterns are in fact perceived to move faster. Additionally, they also found that striped objects were more often caught behind the centre compared with unicolour stimuli and suggested that incorrect speed perception of the stimulus might have caused this paradoxical observation. Therefore, it is likely that the reference stimulus used in speed match experiments might play a crucial role in deciding the outcome of the experiment. However, we note that correlation studies on snakes with longitudinal stripes [3,11,39] indicate that animals having longitudinal striped pattern might be perceived to move slower, indirectly supporting the results of our speed match experiment.

### 4.2 Why do lizards have stripes only on the head and trunk?

One of the proposed mechanisms for dazzle patterns in preventing successful capture is the size of the local receptive field of predators involved in processing visual information, ‘The Aperture problem’ [8]. As the size of the receptive field involved in processing early motion signals in vertebrates is small, it is possible that the accuracy of attacks is affected by the eye gaze being directed to an apparently immobile anterior part of the prey containing striped patterns rather than global prey movement [40]. Moreover, it is known that predators target vital body parts such as the head or trunk to thwart prey escape [41], particularly in the case of lizards [27,28,42]. This was approximated in our experiments by instructing the volunteers to attack the anterior half of the moving prey. Therefore, the non-significant effect of prey type on either *n*_lethal_/*n*_non-lethal_ attacks or total number of hits in Experiment 2 corroborates the prediction that the presence of stripes at the anterior is important for a redirective effect. In particular, many lizards with striped pattern on the body seem to have a conspicuously coloured tail (electronic supplementary material, figure S10). Therefore, it is likely that during motion, the redirective functioning of longitudinal stripes on the body of lizards is amplified by the presence of highly contrasting, conspicuously coloured tail that provides a ‘trackable feature’ [8,44, p. 129].

Although there is mounting evidence for the classical deflection hypothesis where colour patterns or morphological structures on less vulnerable parts *attract* attention *towards* themselves [23,24,43], this is the first study that has shown that patterns on vital anterior body parts can *redirect* attacks *away* from themselves towards the less-vital posterior parts. To distinguish between the two hypotheses, we christen the latter as the ‘redirection hypothesis’. We emphasize that our hypothesis refers specifically to redirection of attacks (i) during motion and (ii) relative to the direction of movement, i.e. predatory attacks are misdirected in a direction opposite to that of prey motion.

## 5. Conclusion

In summary, we have clearly demonstrated that striped patterns on the vital anterior body parts of lizards and similar animals can redirect attacks away from themselves, towards expendable posterior body organs. How this relates to the evolution of other correlated traits, for example, behaviour or morphological traits in natural prey will be an interesting question for future studies. We also show the mechanism behind the redirection effect; stripes achieve the redirective effect by making the prey appear to move slower than in reality. Further work is needed to understand how different kinds of patterns increase or decrease perceived speed and how this alteration of speed perception could be an adaptive strategy. This is the first study that demonstrates that conspicuous patterns can be adaptive by redirecting attacks away from themselves.

## Supplementary Material

1. Supplementary_material_1: Additional results and procedure

## Supplementary Material

2. Supplementary_material_2: Instruction sheet to participants

## Data accessibility

The datasets supporting the results are available online in Dryad repository (http://dx.doi.org/10.5061/dryad.mp089).
