# Supplementary material for: Deceived by stripes: conspicuous patterning on vital anterior body parts can redirect predatory strikes to expendable posterior organs
Source: R Soc Open Sci. 2016 Jun 8;3(6):160057. doi: 10.1098/rsos.160057 (PMC4929900; doi:10.1098/rsos.160057)
Supplement: 1. Supplementary_material_1: Additional results and procedure [file rsos160057supp1.pdf]

INSTRUCTIONS

1. Your objective is to catch the front end of a rectangular object moving randomly on the screen.

Direction of movement

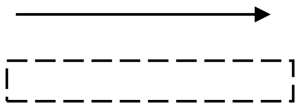

Back end Front end

Direction of movement

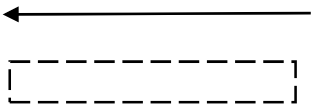

Front end Back end

2. Preferably touch the front end of the object with one of your finger (always use the same) on the monitor.
3. Catch as many objects as you can within the time limit of 1 minute.
4. Position yourself such that the distance between your head and the screen does not change during the game.
5. Please ensure that you sit directly in front of the screen and not to lean toward the monitor screen.

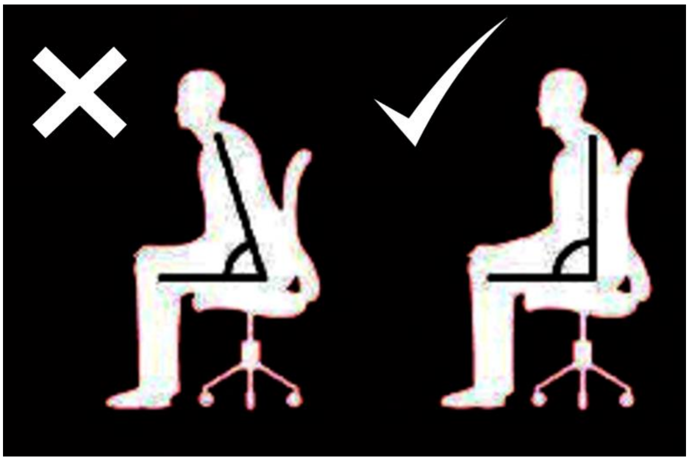

6. The game will end automatically after 1 minute.
7. Please do not discuss the game with others as it may influence the outcome of the experiment.
8. Tell the age group you belong from the table given below.
9. Press the **green flag** at the **top right corner** to **start the game**. 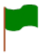

Age group (years)
15-20
20-25
25-30
30-35
35-40
40-45
45-50
50-55
55-60
60-65
