# Supplementary material for: Deceived by stripes: conspicuous patterning on vital anterior body parts can redirect predatory strikes to expendable posterior organs
Source: R Soc Open Sci. 2016 Jun 8;3(6):160057. doi: 10.1098/rsos.160057 (PMC4929900; doi:10.1098/rsos.160057)
Supplement: 2. Supplementary_material_2: Instruction sheet to participants [file rsos160057supp2.pdf]

Supplementary material

Text S1: Details of the experimental design

The game

The experiment was a computer 'game' played on the touch screen monitor of a HP Envy 17.3-Inch Touch-smart laptop by human subjects. The prey object entered the display area from any of the four edges (left, right, top or bottom; chosen randomly) with a random entry angle within the range of -30° to +30° (normalized to the horizontal axis). The object moved within the display area until it touched an edge of the display or was 'attacked'. If the prey touched any edge or if it was caught, it would restart from one of the four randomly chosen edges with a delay of 0.10 second. While moving on the display area, the prey also changed its direction of movement erratically. This was done by allowing it to take on an angle within the range -10° to +10°, with the angle changed after every 0.5 cm displacement. This unpredictable direction change created a 'wobble' appearance mimicking the movement of a lizard and made the prey more difficult to catch. For all the experiments, the game lasted for 1 minute per prey. A plain achromatic grey (R-128, B-128, G-128) image was used as background on the screen for all the experiments.

Scoring the number of hits

A circle of diameter 0.3 cm (0.264 degrees) was used as a 'touch sensor' to record the number of attacks. The number of hits was scored based on the sensor touching different color combinations in the prey. When the sensor touched the color on the anterior part of the prey, it was considered an attack on the anterior and scored as *'anterior'* by increasing the 'Anterior' count variable in the game by 1. Similarly, an attack was scored as '*posterior*' when the sensor touched the color on the posterior half of the object. If sensor touched both *anterior* and *posterior* color combinations, the hit was scored as '*center*' (See **Table S1** below). The count value for the above variables were made invisible to participants during the progress of the experiment.

Volunteers were drawn from the graduate students and faculty of the Indian Institute of Science Education and Research Thiruvananthapuram. Participants were first asked to sign a written consent form in accordance with the Declaration of Helsinki and approved by the Institute's Ethics Committee. All participants were blind to the hypotheses of the experiments and participated voluntarily. Subjects were allowed to terminate the trial at any point without an explanation, and no sensitive information other than subject's name was collected to avoid pseudo-replication. Accumulating evidence suggests that predators aim to attack the most vulnerable body parts to thwart prey escape (1,2), and hence we instructed participants to attack the anterior part of the prey. Each individual was instructed to attack two prey (treatment and control) one after the other. The above design accounts for variation among individuals in terms of attack frequency and 'capture' efficiency.

All experiments were conducted in well-lit laboratory conditions, between 9.30 a.m. to 6 p.m. A viewing distance of 65 cm was maintained for all the experiments. Artificial lighting was controlled to avoid the reflections from the monitor display. An equal number of test subjects were utilized for both the *Sets* of prey and each of the two prey in an experiment was presented first for half of the participants, and second for the other half. No individual was used twice, and all volunteers had normal or vision corrected to normal by glasses or contact lenses.

Speed match experiment

The experiment was conducted using the same laptop as used in the first experiment and with same display settings. The stimuli entered the display area from the bottom edge with a random entry angle within the range of -30° to +30° (normalized to the horizontal axis), in order to avoid speed judgments based on the distance travelled by the prey or time taken for disappearance. The stimulus was allowed to move within the display until it touched the edge. Once it touched an edge, it respawned again at the bottom of the display. An auditory tone was played before the appearance of stimuli on the screen. The background used was the same as in the previous experiments. Participants observed the moving stimuli (S-A and C) displayed sequentially from a fixed distance of ca. 65 cm.

Each stimulus appeared from the bottom edge and disappeared once it reached any other edge. Immediately after this, the same stimulus appeared again from the bottom edge and disappeared upon reaching another edge. After two such appearances/disappearances of the first stimulus, there was an interval of 1.5 seconds after which the next stimulus appeared. As with the first stimulus, the second stimulus also appeared /disappeared twice. Each stimulus was made to appear/disappear twice because our pilot studies showed that it was difficult to judge the speed based on only one appearance/disappearance. The order of presentation of the stimuli was randomized. Participants indicated their response by touching the appropriate stimulus containing the pattern or equal symbol in the question window (**Figure S9**)

Table S1. Scoring of the number of hits. For illustration purposes, prey from experiment 1 are shown (See **Text S1** above for details)

Experiment	Set number	Object type	Colour combination touched	Location scored
1	1	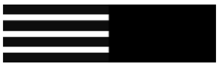	(Greyish-black & White) or (Greyish-black) or (White)	Anterior
			Black	Posterior
			(Greyish-black & Black) or (White & Black)	Centre
		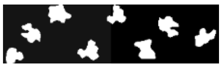	(Greyish-black & White) or (Greyish-black)	Anterior
			(Black & White) or (Black)	Posterior
			Greyish-black & Black	Centre
	2	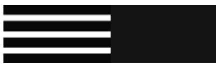	(Black & White) or (Black) or (White)	Anterior
			Greyish-black	Posterior
			(Greyish-black & Black) or (White) & (Greyish-black)	Centre
		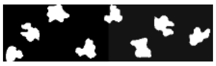	(Black & White) or (Black)	Anterior
			(Greyish-black & White) or (Greyish-black)	Posterior
			(Greyish-black & Black)	Centre

*A circle of diameter 0.3 cm was used as a sensor, which is larger than the size of any single blotch or width of a stripe.

Table S2: Results of the Poisson GLMM analysis with lethal attacks (*anterior* + *centre*) as dependent variable and presentation order, prey type and interaction between prey type and presentation order as fixed effects. *Set* number and Subject ID were included as random intercept terms.

Experiment 1:
Results of best fit GLMM model:

**Factor**	**Estimate**	**Standard error**	**Z value**	**P-value**
Intercept	3.17936	0.06027	52.76	< 0.001
Prey type	-0.23473	0.06322	-3.71	0.000205
Presentation order	0.11440	0.08227	1.39	0.164376
Prey type: Presentation order	-0.25168	0.08829	-2.85	0.004365

Experiment 2: (No model was significantly better than another)
Results from model selection procedure (Likelihood ratio test):

**Factor**	**Degrees of freedom**	**AIC**	**AIC weights**	**Chi square value**	**P-value**
Null model	3	693.19	0.2663	-	-
Lethal_attacks ~ Prey type	4	692.84	0.3173	2.3444	0.1257
Lethal_attacks ~ Presentation order	5	693.09	0.2800	1.7490	0.1860
Lethal_attacks ~ Prey type * Presentation order	6	694.53	0.1363	0.5686	0.4508

Experiment 3:
Results of best fit GLMM model:

**Factor**	**Estimate**	**Standard error**	**Z value**	**P-value**
Intercept	3.13211	0.05964	52.52	< 0.001
Prey type	-0.17452	0.06229	-2.80	0.00508
Presentation order	0.09685	0.08235	1.18	0.23955
Prey type: Presentation order	-0.26731	0.08848	-3.02	0.00252

Table S3: Results of the Poisson GLMM analysis with non-lethal attacks (*posterior*) as dependent variable and presentation order, prey type and interaction between prey type and presentation order as fixed effects. *Set* number and Subject ID was included as random intercept terms. The results of best fit model are reported below.

Experiment 1:
Results of the best fit GLMM model:

**Factor**	**Estimate**	**Standard error**	**Z value**	**P-value**
(Intercept)	2.2985	0.0877	26.192	<0.0001
Prey type	0.6057	0.0800	7.563	<0.0001
Presentation order	0.41084	0.1158	3.547	0.0003
Prey type: Presentation order	-0.2630	0.1038	-2.534	0.0112

Experiment 2: (No model was significantly better than another)
Results from model selection procedure (Likelihood ratio test):

**Factor**	**Degrees of freedom**	**AIC**	**AIC weights**	**Chi square value**	**P-value**
Null model	3	682.12	0.4591	-	-
Non-lethal_attacks ~ Prey type	4	683.69	0.2094	0.4267	0.1257
Non-lethal_attacks ~ Presentation order	5	685.08	0.1045	0.6112	0.1860
Non-lethal_attacks ~ Prey type* Presentation order	6	683.53	0.2268	3.5558	0.4508

Experiment 3:
Results of the best fit GLMM model:

**Factor**	**Estimate**	**Standard error**	**Z value**	**P-value**
(Intercept)	2.76371	0.05933	46.58	<0.0001
Prey type	0.28125	0.04610	6.10	<0.0001

Table S4: Summary of possible contrasts for interaction between prey type and presentation order for total number of hits from Experiment 1 and Experiment 2. Contrasts represents *Prey type (Presentation order: R-control; S- stripes)*

Experiment	Contrasts	Z ratio	P-value
Experiment 1	Control (R) – Stripes (R)	-1.9733	0.1982
	Control (R) – Control (S)	-3.5283	0.0024*
	Control (R) – Stripes (S)	-1.7556	0.2951
	Stripes (R) – Control (S)	-1.9388	0.2118
	Stripes (R) – Stripes (S)	-0.1599	0.9985
	Control (S) –Stripes (S)	2.3434	0.0885
Experiment 3	Control (R) – Stripes (R)	-1.5172	0.4271
	Control (R) – Control (S)	-1.3669	0.5202
	Control (R) – Stripes (S)	0.4151	0.9759
	Stripes (R) – Control (S)	-0.3371	0.9868
	Stripes (R) – Stripes (S)	1.4455	0.4709
	Control (S) –Stripes (S)	2.6808	0.0370*

Table S5: Results of the GLMM analysis for across experiment comparisons for control prey (C *vs* C-A). The main model included experiment number and presentation order as fixed effects and *Set* number as random intercept term with counts of n_{lethal} and $n_{non\text{-}lethal}$ as binomial dependent variable (using cbind) with logit link function. The interaction between presentation order and experiment was not included in the main model as it did not significantly improve the fit of the model (LRT: X^2= 5.6152, p=0.0635). The results of *tukey* post hoc multiple contrasts calculated using *multcomp* package (3) are presented below. Refer to **Supplementary figure S3** for more details.

Factors	Estimate	Standard Error	Z-value	P-value
Experiment 1 *vs* Experiment 3	-0.30885	0.06679	-4.624	<0.001
Experiment 1 *vs* Experiment 2	0.01998	0.06849	0.292	0.954
Experiment 3 *vs* Experiment 2	0.32882	0.06570	5.005	< 0.001

Table S6: Results of the GLMM analysis for across experiment comparisons for striped prey (S-A *vs* S-P). The main model included experiment number and presentation order as fixed effects and *Set* number as random intercept term with counts of n_{lethal} and $n_{non\text{-}lethal}$ as binomial dependent variable (using cbind) with logit link function. The interaction between presentation order and experiment was not included in the main model as it did not significantly improve the fit of the model (LRT: $X2$=1.5802 p= 0.4537). The results of *tukey* post hoc multiple contrasts were calculated using *multcomp* package (3) is presented below. Refer **Supplementary figure S4**

**Factors**	**Estimate**	**Standard Error**	**Z-value**	**P-value**
Experiment 1 *vs* Experiment 3	-0.07939	0.06508	-1.22	0.441
Experiment 1 *vs* Experiment 2	0.87259	0.06603	13.21	<0.001
Experiment 2 *vs* Experiment 3	-0.95198	0.06504	-14.64	< 0.001

Table S7: Stepwise model selection for binomial GLMM on lethal *vs* non-lethal attacks

Dependent Variable (DV)- cbind (n_{lethal}, $n_{non\text{-}lethal}$)

The best fit model reported in the main text is highlighted in bold and underlined. The model estimates of the best fit model are reported in the main text **Table 1**.

(a) Experiment 1

Model formula	Degrees of freedom	AIC	AIC weights	Chi-square	P-value
DV ~ Prey type * Presentation order	6	563.63	0.2147	0.0119	0.9130
**<u>DV ~ Prey type + Presentation order</u>**	**<u>5</u>**	**<u>561.64</u>**	**<u>0.5809</u>**	**<u>4.0957</u>**	**<u>0.0429</u>**
DV ~ Prey type	4	563.73	0.2043	159.5756	<0.0001
DV ~1	3	721.31	<0.0001	-	-

(b) Experiment 2 (No model was significantly better than another)

Model formula	Degrees of freedom	AIC	AIC weights	Chi-square	P-value
DV ~ Prey type * Presentation order	6	629.32	0.1259	1.5050	0.2199
DV ~ Prey type + Presentation order	5	628.82	0.1617	1.5113	0.2189
DV ~ Prey type	4	628.33	0.2066	0.2091	0.6475
DV ~1	3	626.54	0.5056	-	-

(c) Experiment 3

Model formula	Degrees of freedom	AIC	AIC weights	Chi-square	P-value
DV ~ Prey type * Presentation order	6	603.19	0.1519	1.0309	0.3099
DV ~ Prey type + Presentation order	5	602.22	0.2468	0.2213	0.6381
**<u>DV ~ Prey type</u>**	**<u>4</u>**	**<u>600.44</u>**	**<u>0.6011</u>**	**<u>99.4519</u>**	**<u><0.0001</u>**
DV ~ 1	3	697.90	<0.0001	-	-

Table S8: Model selection for Poisson GLMM on total number of hits

Dependent Variable (DV)- total number of hits (*anterior +centre + posterior*)

The best fit model is highlighted in bold and underlined. The estimates of the best fit model are reported in **Supplementary Table S9**.

(a) Experiment 1

Model formula	Degrees of freedom	AIC	AIC weights	Chi-square	P-value
**<u>DV ~ Prey type * Presentation order</u>**	**<u>6</u>**	**<u>665.51</u>**	**<u>0.9506</u>**	**<u>9.2121</u>**	**<u>0.0024</u>**
DV ~ Prey type + Presentation order	5	672.72	0.0258	4.6876	0.0303
DV ~ Prey type	4	675.41	0.0067	0.1673	0.6824
DV ~ 1	3	673.58	0.0168	-	-

(b) Experiment 2 (No model was significantly better than another)

Model formula	Degrees of freedom	AIC	AIC weights	Chi-square	P-value
DV ~ Prey type * Presentation order	6	743.96	0.2332	2.9142	0.0878
DV ~ Prey type + Presentation order	5	744.88	0.1472	0.2144	0.6433
DV ~ Prey type	4	743.09	0.3603	2.6576	0.1031
DV ~1	3	743.75	0.2590	-	-

(c) Experiment 3

Model formula	Degrees of freedom	AIC	AIC weights	Chi-square	P-value
**<u>DV~ Prey type *Presentation order</u>**	**<u>6</u>**	**<u>724.39</u>**	**<u>0.7676</u>**	**<u>8.7445</u>**	**<u>0.0031</u>**
DV~ Prey type+ Presentation order	5	731.14	0.0262	0.0008	0.9768
DV~ Prey type	4	729.14	0.0713	0.7298	0.3929
DV~1	3	727.87	0.1347	-	-

Table S9: Results of Poisson GLMM for the best fit model on total number of hits

Dependent Variable (DV)- Total number of hits (*anterior* + *centre* + *posterior*)

(a) Experiment 1:

**Factor**	**Estimate**	**Standard error**	**Z value**	**P-value**
Intercept	3.5454	0.0441	80.36	< 0.0001
Prey type	0.0961	0.0487	1.97	0.04845
Presentation order	0.2091	0.0592	3.53	0.00041
Prey type: Presentation order	-0.1997	0.0657	-3.04	0.00239

(b) Experiment 3:

**Factor**	**Estimate**	**Standard error**	**Z value**	**P-value**
Intercept	3.65975	0.04795	76.33	< 0.0001
Prey type	0.06857	0.04519	1.52	0.12920
Presentation order	0.09078	0.06641	1.37	0.17165
Prey type: Presentation order	-0.18728	0.06327	-2.96	0.00308

Table S10: Model selection for LMM for speed match experiment (Experiment 4)

Dependent Variable (DV)- Matched speed

The best fit model is highlighted in bold and underlined. The estimates of the best fit model are reported in **Supplementary Table S11**.

(a) Experiment 4 (i) Control (C) as standard

Model formula	Degrees of freedom	AIC	AIC weights	Chi-square	P-value
DV ~ Prey type * Initial speed	8	845.16	0.0540	0.8846	0.6426
DV ~ Prey type + Initial speed	6	842.04	0.2572	2.0334	0.3618
**<u>DV ~ Prey type</u>**	**<u>4</u>**	**<u>840.07</u>**	**<u>0.6887</u>**	**<u>23.2590</u>**	**<u><0.0001</u>**
DV ~ 1	3	861.33	<0.0001	-	-

(b) Experiment 4- (ii) Striped prey (S-A) as standard

Model formula	Degrees of freedom	AIC	AIC weights	Chi-square	P-value
DV ~ Prey type *Initial speed	8	770.05	0.0229	0.3132	0.8550
DV ~ Prey type + Initial speed	6	766.36	0.1452	0.5114	0.7744
**<u>DV ~ Prey type</u>**	**<u>4</u>**	**<u>762.87</u>**	**<u>0.8317</u>**	**<u>30.3720</u>**	**<u><0.0001</u>**
DV ~ 1	3	791.24	<0.0001	-	-

REFERENCES:

1. Vervust B, Loy H, Damme R. Seeing through the lizard's trick: do avian predators avoid autotomous tails? Open Life Sci. 2011;6(2):293–9.

2. Fouts WR, Nelson DR. Prey capture by the Pacific angel shark, Squatina californica: visually mediated strikes and ambush-site characteristics. Copeia. 1999;304–12.

3. Hothorn T, Bretz F, Westfall P. Simultaneous inference in general parametric models. Biom J Biom Z. 2008 Jun;50(3):346–63.

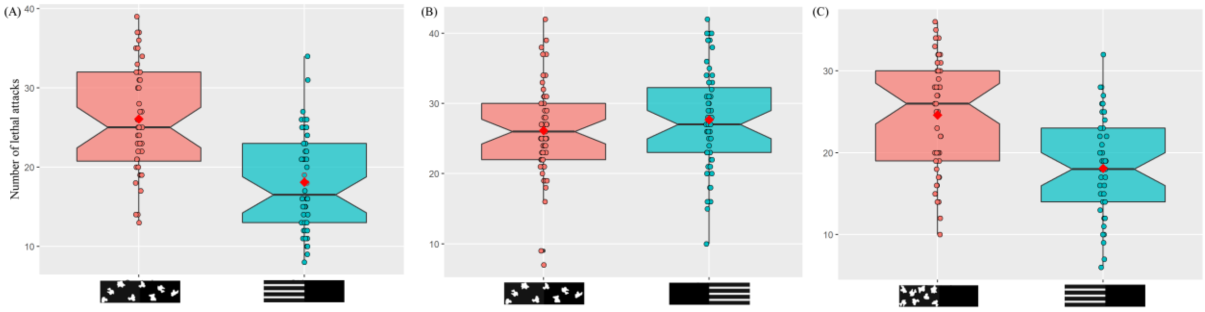

Figure S1: Boxplots showing the number of lethal attacks (*anterior* + *centre*) received by prey in: (A) Experiment 1 (B) Experiment 2 (C) Experiment 3; Refer to Supplementary **Table S2** for statistical results.

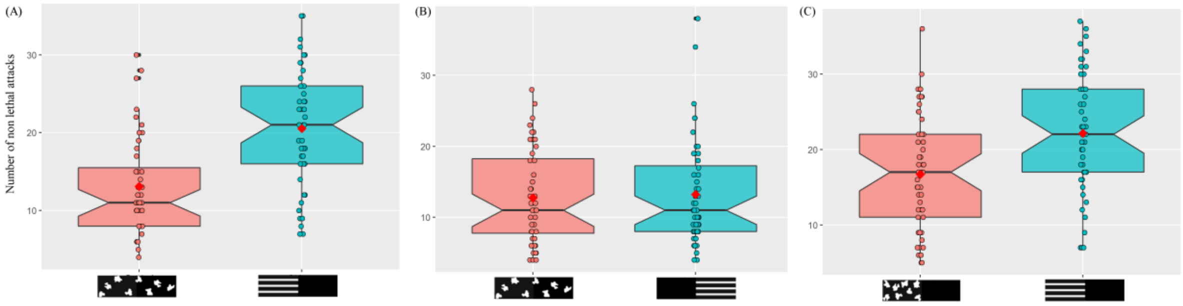

Figure S2: Boxplots showing the number of non-lethal attacks (*posterior*) received by prey in: (A) Experiment 1 (B) Experiment 2 (C) Experiment 3; Refer to Supplementary **Table S3** for statistical results.

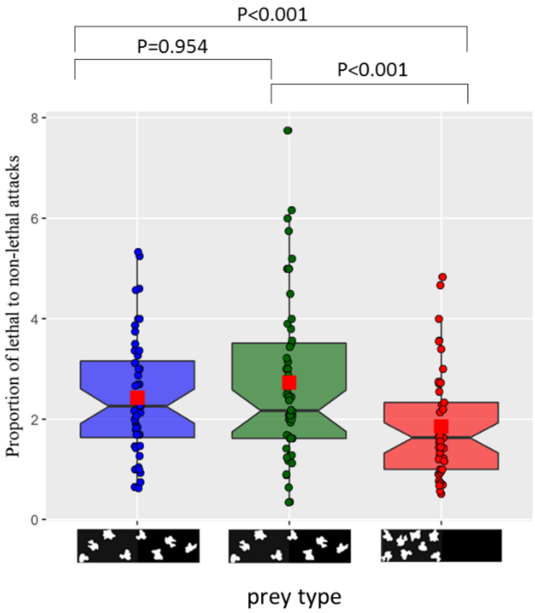

Figure S3: Boxplots showing the proportion of lethal (*anterior* + *centre*) to non-lethal attacks (*posterior*) for control objects across experiments: Prey from Experiment 3 (C-A) received lower $n_{lethal}/n_{non\text{-}lethal}$ attacks compared to controls (C) from both Experiment 1 (z=-4.624, p<0.001) and Experiment 2 (z=5.005, p<0.001). However, there was no difference in $n_{lethal}/n_{non\text{-}lethal}$ attacks between Experiment 1 and Experiment 3 (z=0.0292, p=0.954); refer to **Table S4** for a detailed summary

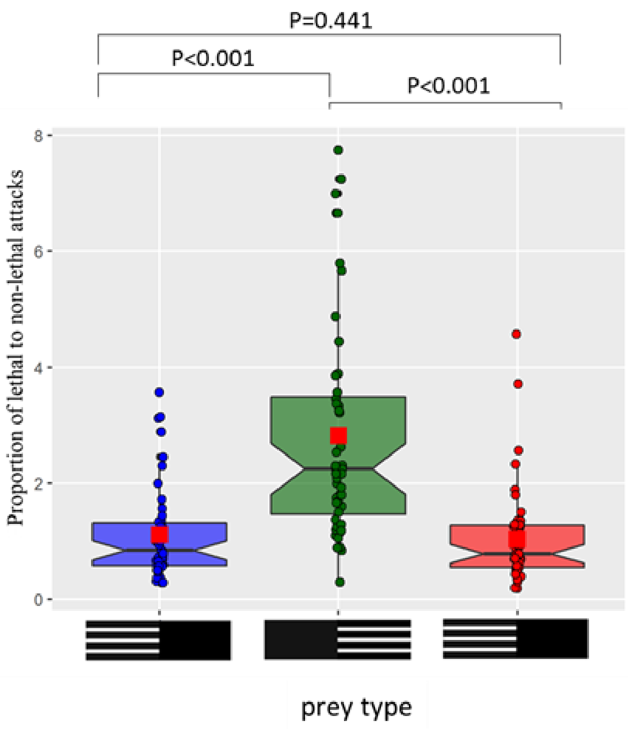

Figure S4: Boxplots showing the proportion of lethal (*anterior* + *centre*) to non-lethal attacks (*posterior*) for striped prey across experiment: Prey from Experiment 2 (S-P) received higher $n_{lethal}/n_{non\text{-}lethal}$ attacks when compared to striped prey (S-A) from both Experiment 1 (z=13.21, p<0.001) and Experiment 3 (z=-14.64, p<0.001). However, there was no difference in $n_{lethal}/n_{non\text{-}lethal}$ attacks between Experiment 1 and Experiment 3 (z=1.5802 p= 0.4537); refer to **Table S6** for a detailed summary.

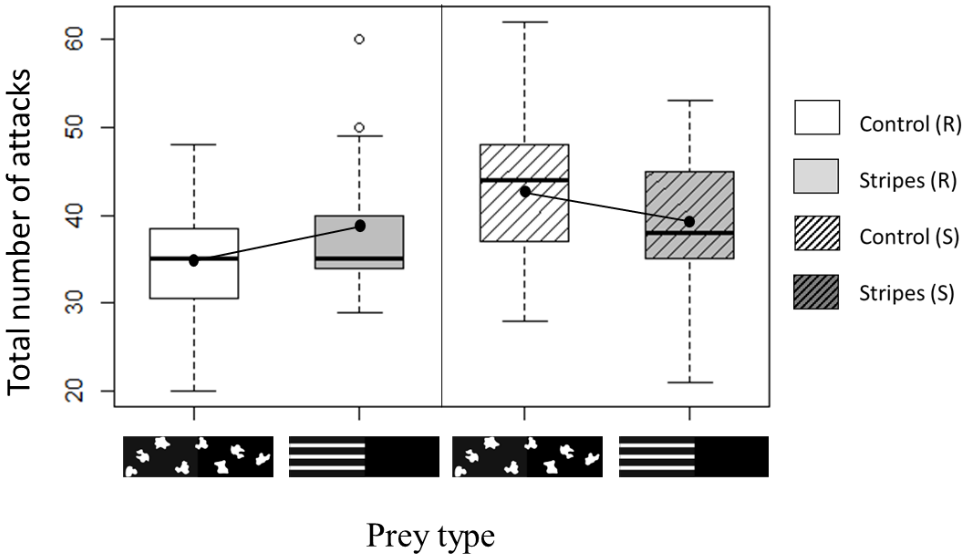

Figure S5: Boxplot showing the interaction between object type and presentation order in Experiment 1: Boxes with stripes indicate data when striped prey (S-A) was presented first (S) and boxes without stripes indicate when control (C-A) was presented first (R). Means are represented by filled black circles. The Control (R) received a significantly lesser number of total hits than Control (S) prey (*z* ratio= -3.5283, p=0.0024), other pairwise contrasts are not significantly different; refer to **Table S4** for a detailed summary.

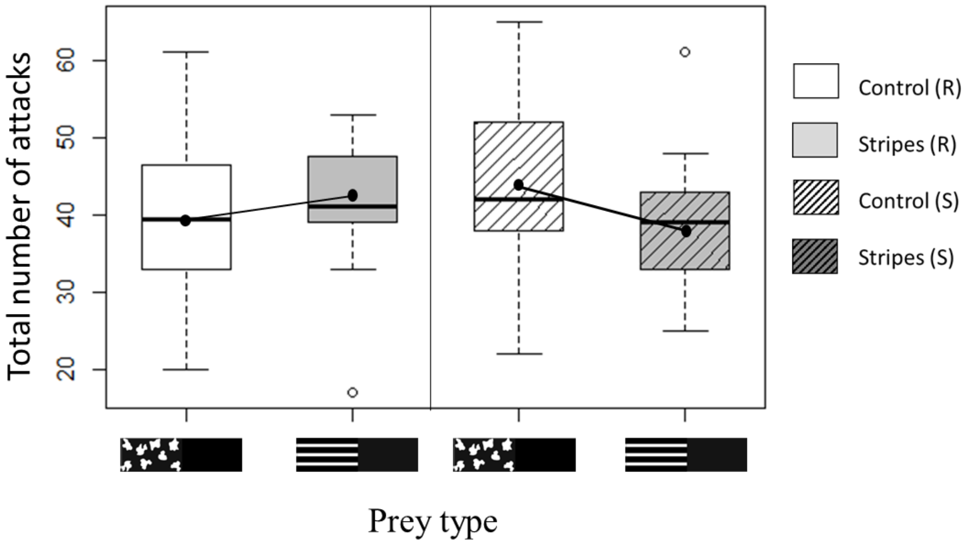

Figure S6: Boxplot showing the interaction between object type and presentation order in Experiment 3: Boxes with stripes indicate data when striped prey (S-A) was presented first (S) and boxes without stripes indicate when control (C-A) was presented first (R). Means are represented with black filled circles. Control (s) received a significantly greater number of total hits than Stripes (S) prey (*z* ratio=2.6808, p=0.0370), other pairwise contrasts are not significantly different; refer to **Table S4** for a detailed summary.

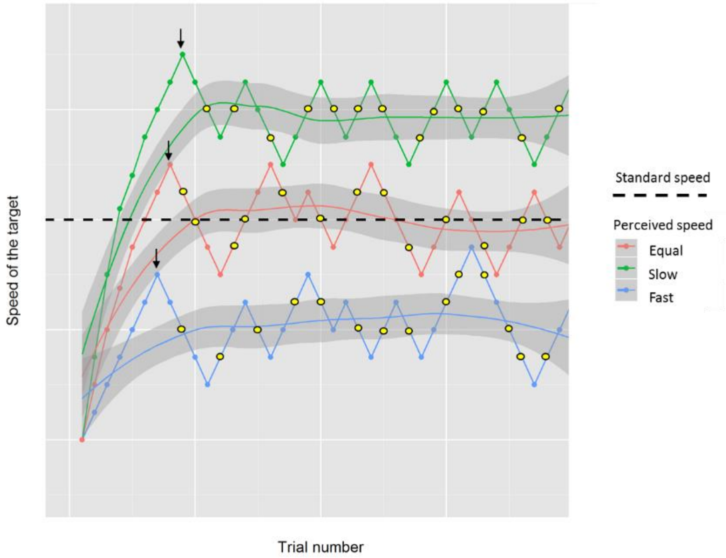

Figure S7: Graphical illustration of possible results in a speed match experiment. The speed of the target versus trial number for a single person is plotted. For simplicity, all illustrations were shown for initial speed of target being lower than that of the standard. **Condition 1** (green): perceived speed of target is slower than standard, **Condition 2** (red): perceived speed of target is equal to standard, **Condition 3** (blue): perceived speed of target is faster than standard. Yellow solid circle represents hypothetical speed values at which both standard and target stimuli are perceived to move at same speed which was ultimately considered for the analysis. Arrows represent first reversal events.

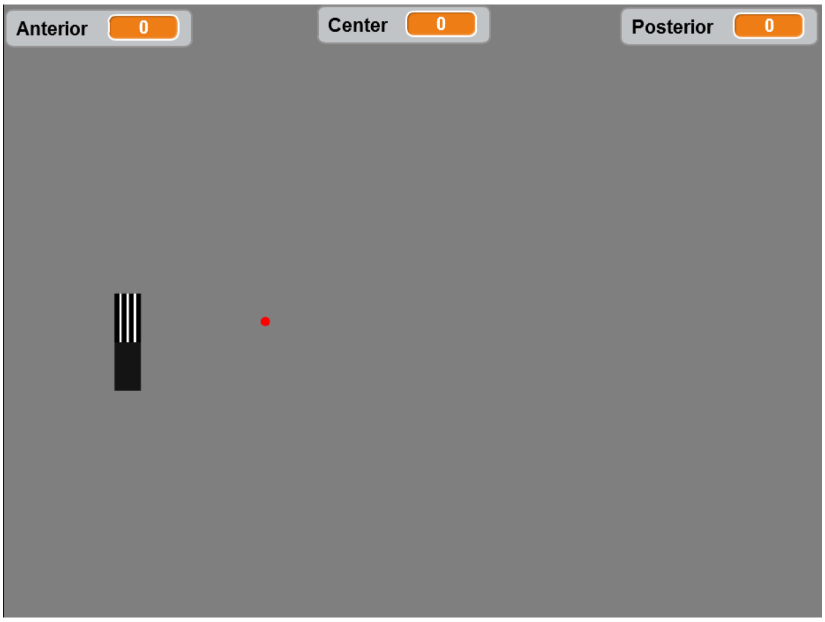

Figure S8: Screenshot of the experimental set-up with different variables recorded during the experiment (Prey size not to scale). Note that the variables were not visible to the subjects.

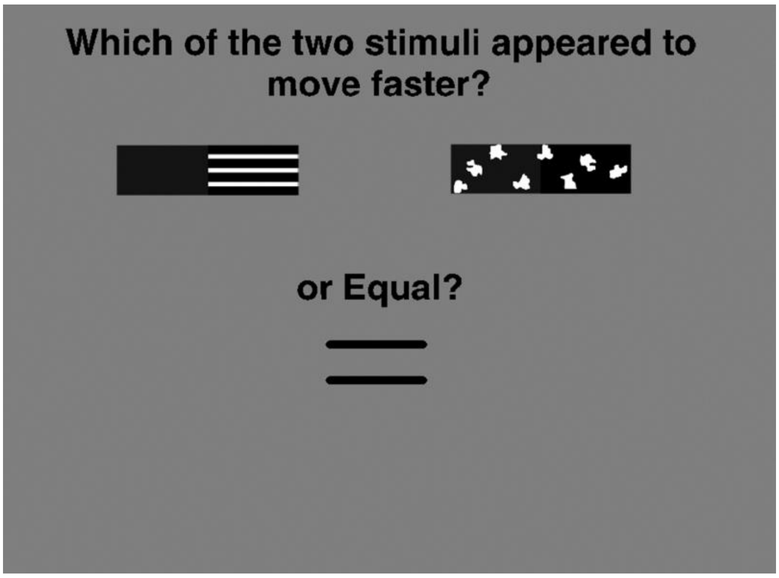

Figure S9: Screenshot of the question window used in speed match experiment.

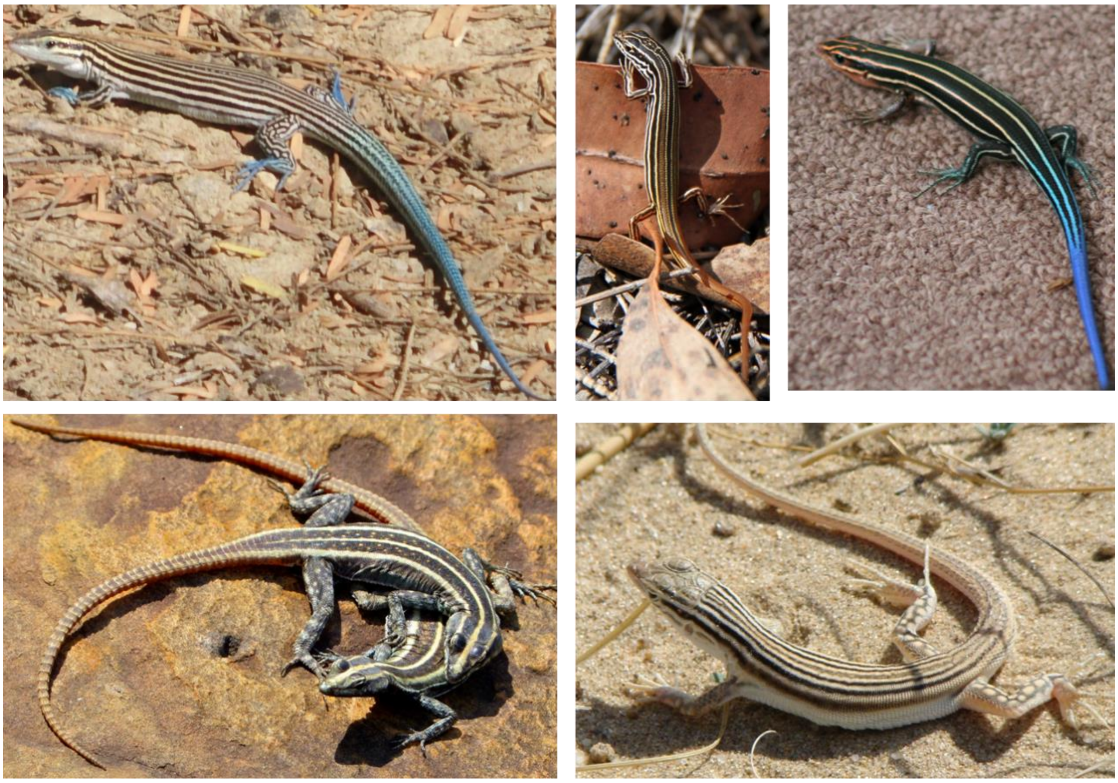

Figure S10: Example of lizard species with striped coloration from different families: Top left: *Ameiva lineolate* - Teiidae (photo credit: Martin Reith); Top middle: *Ctenotus taeniolatus* - Scincidae (photo credit: Greg Schechter); Top right: *Plestiodon fasciatus* - Scincidae (photo credit: Eric Fleming); Bottom left: *Platysaurus intermedius* - Cordylidae (photo credit: Bernard DUPONT) Bottom right: *Eremias lineolate* - Lacertidae (photo credit: Yuriy75). All photographs were accessed via Wikimedia commons.
